# Highly diversified multiply drug-resistant HIV-1 quasispecies in PBMCs: a case report

**DOI:** 10.1186/1742-4690-5-43

**Published:** 2008-05-30

**Authors:** Yudong Quan, Bluma G Brenner, André Dascal, Mark A Wainberg

**Affiliations:** 1 McGill University AIDS Centre, Lady Davis Institute-Jewish General Hospital, 3755 Cote Ste-Catherine Road, Montreal, Quebec, H3T 1E2, Canada

## Abstract

**Background:**

Although drug resistance is a major challenge in HIV therapy, the effect of drug resistance mutations on HIV evolution in vivo is not well understood. We have now investigated genetic heterogeneity in HIV-1 by performing drug resistance genotyping of the PR-RT regions of viruses derived from plasma and peripheral blood mononuclear cells (PBMCs) of a single patient who had failed multiple regimens of anti-retroviral therapy.

**Results:**

Patterns of drug resistance mutations showed that the viral populations in PBMCs were more heterogeneous than in plasma. Extensive analysis of HIV from infected PBMCs in this patient showed that high-level diversity existed among 109 cloned PR-RT sequences and that the majority of mutations were related to drug resistance. Moreover, the PBMCs included archival species that reflected the treatment history of the patient while those in plasma were mainly related to the most recent treatment. Some of the proviral clones contained single or multiple mutations in various combinations. Approximately eighteen percent of the proviral clones derived from infected PBMCs were defective, i.e. 5.5% contained single nucleotide deletions (frameshift mutations) and 12.8% encoded in-frame stop codons (nonsense mutations). Amino acid substitutions in PR and the polymerase region of RT occurred in 12–15% of cases but were much less frequent in the RNase H region of RT, which might not have been under drug selection pressure.

**Conclusion:**

Selective drug pressure can yield multiple drug-resistant quasispecies that include archival and replication-incompetent species in PBMC reservoirs.

## Findings

HIV quasispecies within infected individuals can rapidly adapt to hosts [1-7] due, in part, to variations in replicative fitness that enable some viruses to grow faster than others[3,8]. This is of obvious clinical relevance, since viral genetic changes can result in alterations in receptor usage, escape from drug and host immune pressure, and can impact on viral pathogenesis[9]. HIV-1 may also evolve separately in different physiological compartments, e.g., peripheral blood mononuclear cells (PBMCs) vs. the central nervous system[10]. Here, we report on an individual who failed multiple antiviral therapies (ART), including use of nucleoside and non-nucleoside RT inhibitors (NRTIs and NNRTIs) and protease inhibitors (PIs). After initiating therapy elsewhere with undisclosed regimens, the patient was treated in 1999 at the Jewish General Hospital, Montreal, Canada, with zidovudine (AZT)/lamivudine (3TC)/efavirenz (EFV) plus unboosted indinavir (IDV) and nelfinavir (NFV) for 9 months and 3 months, respectively, and was switched to stavudine (d4T)/3TC/amprenavir (APV) for 12 months, at which time viral samples were obtained for resistance testing. Viral RNA from plasma and proviral DNA from PBMCs were purified using commercial kits (Qiagen, Mississauga, ON, Canada). Initial HIV-1 genotyping was performed using Trugene HIV-1 genotyping kits (Siemens Diagnostics Inc., Toronto, Canada). All studied were performed with approval of the Ethics Review Committee, Jewish General Hospital.

### The degree of quasispecies heterogeneity was higher in PBMCs than in plasma

Mutations in PR and RT associated with drug resistance were compared in plasma vs PBMCs. Both types of samples contained viruses with multiple primary (M46I/L, G48V, I54V, V82A or L90M) and secondary resistance mutations (e.g. L10I) in PR as well as multiple mutations in RT (M41L, E44A, T69N, V118I, M184V, L210W, T215Y, K219R for NRTIs) (A98G, K101E, V1081, Y181C and G190A for NNRTIs) (Table 1). Both the plasma and PBMC samples contained mixtures of mutations, although some mutational motifs were only detected in the PBMCs. For example, mixtures of 41K/R, 54I/V, 64I/V, 82V/A, 90M/I in PR and 181Y/C, 190A/G, 219K/R in RT were identified in PBMCs but not in plasma. Conversely, 35D and 69N in PR and 108I in RT were detected only in plasma but not PBMCs, as determined by genotyping. These results were confirmed by clonal sequencing of PBMC DNA. In general, viruses harbouring the unboosted protease motif including L90M were exclusively present in PBMCs. This is consistent with the fact that genotyping often fails to detect minority species that are represented at levels <10 to 35% in a given population [11].

**Table 1:** Comparisons of plasma and PBMC genotypes of the PR and RT regions in the HIV-1 infected patient Position in PR or RT^a^

**PR**	**10**	**15**	**16**	**35**	**37**	**39**	**41**	**46**	**48**	**54**	**60**	**62**	**63**	**64**	**68**	**69**	**72**	**77**	**82**	**90**					
**Plasma**	I	I/V	E	D	T	S		L	V	V	E	V	P/H	V		H/Y	V	I	V/A						
**PBMCs**	I		E		T	S	K/R	I/M	V	I/V	E	V	P	I/V	G/R	H/Y	V	I	V/A	M/I					
**RT**	**41**	**43**	**44**	**69**	**98**	**101**	**102**	**108**	**118**	**122**	**162**	**166**	**177**	**181**	**184**	**190**	**203**	**208**	**210**	**211**	**214**	**215**	**219**	**223**	**228**
**Plasma**	L	E	A	N	G	K/E	Q	I	I	K	D	R	E	C	V	A	K	Y	W	E	F	Y	R	Q	R
**PBMCs**	L	E	A		G	K/E	Q		I	K	H/D	K/R	E	Y/C	V	A/G	K	Y	W	D	F	Y	K/R	Q	R

a: cDNA containing the entire PR and half of RT was synthesized from HIV-1 RNA in plasma by RT-PCR or DNA directly amplified by PCR from proviral DNA of infected PBMCs. DNA was doubly sequenced, visualized and analyzed by the Siemens Automatic System. The sequence of the HIV-1 HxB2 strain was used as the wild type reference strain.

### Resistance-associated mutations in PR-RT clones reveal heterogeneous viral populations within infected PBMCs

Viral genetic diversity in the infected PBMCs was analyzed by randomly selecting and sequencing 109 clones of two independent cloning efforts. Nested PCR was performed to amplify the entire PR-RT region. One pair of primers, forward 5'-ACTGAGAGACAGGCTAATTTTTTAGG and backward 5'-TTGGGCCTTATCTATTTCCAT (Bio S&T, Montreal, Canada) was used for the first round of PCR using *Taq *polymerase (Invitrogen, Burlington, ON, Canada) with 30 cycles of annealing at 55°C for 1 min and extension at 72°C for 3 min. A second round of 25 cycle PCR with primers (forward 5'-ACTATCCATGGTCCCTCAGATCACTCTTTGG and backward 5'-ACTAATTTGTCGACTTGTTCATTTCCTCC (Bio S&T)) was used to generate a 2.1 kb DNA fragment spanning the PR and RT genes. The sample was diluted 100 fold in the second round PCR. The fragment was cloned into the Nco 1-Sal I sites of a modified version of vector pTWIN2 (New England Biolabs, Toronto, Canada) by standard molecular cloning methods. Positive clones were amplified and sequenced using ABI fluorescence sequencing kits (Applied Biosystems, Foster City, CA, USA). Analysis of DNA sequences and protein translation were performed with GeneTool software (BioTools Inc., Alberta, Canada). All viral nucleotide positions were assigned based on differences from the HxB2 reference consensus sequence.

No wild-type (wt) PR or RT sequences were detected in any of these clones, each of which contained a minimum of 8 and a maximum of 12 mutations in PR and a minimum of 11 or a maximum of 19 mutations in RT. A total of 15 mutations were present at resistance-associated sites in PR among the 109 clones tested, while mixtures existed at 9 of these sites. In regard to RT, a total of 19 resistance mutations were detected, of which 11 were also present within mixtures. The frequency of resistance motifs in PBMCs are shown in Table 2. Thymidine analog mutations (TAMs, M41L, L210W, T215Y) were present in all clones while 184V was detected in 81% of sequences. Two reasons may explain the difference. First, the patient had used thymidine analogues for a longer period than 3TC. Second, viruses carrying TAMs are more fit than those with M184V, and the latter mutation may have been under selection pressure even in the presence of 3TC.

**Table 2:** Frequencies of resistance mutations detected in cloned PBMCs^a^

**PR**	**10**	**16**	**35**	**36**	**37**	**41**	**46**	**48**	**54**	**60**	**63**	**69**	**77**	**82**	**90**				
**HxB2**	L	G	E	M	S		M	G	I	D	L	H	V	V	L				
**Clones**	I 100%	E 100%	D 26.0%	V 1%	T 99.0%	K 72.9%	I 42.7%	V 100%	V 54.2%	E 100%	P 99.0%	Y 26.0%	I 100%	A 54.2%	M 45.8%				
				T 1%	I 1.0%		L 55.2%				H 1.0%								
				I 1%			S 1.0%												
**RT**	**41**	**44**	**69**	**74**	**98**	**101**	**102**	**108**	**118**	**181**	**184**	**190**	**208**	**210**	**211**	**214**	**215**	**219**	**283**
**HxB2**	M	E	T	L	A	K	K	V	V	Y	M	G	H	L	R	L	T	K	L
**Clones**	L	A	N	V	G	E	Q	I	I	C	V	A	Y	W	D	F	Y	R	I
	100%	97.9%	12.5%	2.1%	100%	18.75%	97.9%	19.8%	100%	45.8%	81.2%	45.8%	100%	100%	97.9%	100%	100%	47.9%	100%
		T	A			Q	R					T			E				
		2.1%	1.0%			1.0%	21.%					1.0%			2.1%				

a: The PR and RT genes of HIV-1 were amplified by nested PCR and cloned in E coli. The sequences of the clones were determined by BigDye dideoxyterminator sequencing. HxB2 was used as a wild type reference.

ART apparently influenced the emergence of multiple resistance-associated quasispecies. Several PR secondary resistance mutations were found in all of the quasispecies, e.g. 10L, 16E, 37T, 48V, 60E, 63P, 77I. Other mutations were found in only a portion of the PR sequences analyzed, i.e. 35D, 41K, 46I/L, 54V 69Y, 82A and 90M, compared with the HxB2 reference sequence, while the other viruses sequenced were wt in regard to these positions. The 82A and 90M mutations in PR were not found together in any of the clones; neither were 35D and 41K in PR, reflecting the fact that the switch from unboosted to boosted PR inhibitors in the regimen selected for the 82A mutational pathway as opposed to 90M, that represented an archival species present in PBMCs rather than plasma. Resistance-associated substitutions in RT were also found in multiple combinations. 41L, 44A, 98G, 118I, 208I, 210W, 211R, 214F, 215Y, and 283I were found in all the clones (Tables 2 &3). K101E/V108I and Y181C/G190A were mostly present in paired fashion. The frequencies of resistance-associated single substitutions that were only detected in some quasispecies within RT were 74V, 108I, 181C, 184V, and 219R. Several sites contained two or three different amino acid substitutions, i.e., 37T/37S, 46I/46L/46S, 63P/63H within PR and 44A/44T, 69N/69A, 10lE/101Q, 102Q/102R, 190A/190T, 210W/210R/210C, 211D/211E within RT (Table 2). These mixtures may reflect a change from AZT-to d4T-containing regimens. PR had the highest variations of all the sequences analyzed. The average pairwise nucleotide distances of the viral quasispecies within the PBMCs of this patient were 0.022(0.024) for PR, 0.015 (0.012) for the N-terminal 220 amino acids of RT, 0.011(0.007) for the second part of RT (amino acids 221–440) and 0.015(0.013) for RNase H by Mega package (version 4.0) [12]. Numbers in brackets were obtained using the first and second nucleotides of each codon.

**Table 3:** Assignment of clones from infected PBMCs into groups and subgroups based on mutational patterns

Group	Mutational pattern (% representation)	Subgroup	Predominant mutations In PR partially mutated sites	Predominant mutations In RT		Frequency (%)
					
				100% mutated sites	Partially mutated sites	
A	PR90M/46I (40.4)	1		41L/43E/44A/98G/118I/208Y/210W/214F/215Y/228R/283I	181C/190A/219R	4.6
		2	41K/46I/90M		181C/184V/190A/219R	4.6
		3	41K/46I/90M		184V/190A/219R	1.8
		4	41K/46I/90M		184V	18.3
		5	41K/46I/90M		101E/Q/108I/184V	2.8
		6	41K/46I/90M		101E/Q/108I/181C/184V/190A	1.8
		7	36T(V)/41K/46I/90M		184V	1.8
		8	35D/46I/90M		181C/190A/219R	0.9
		9	35D/46I/90M		184V	3.7

B	PR82A/46L/54V/64V (45.9)	10	41K/46V/54V/64V/82A	41L/43E/44A/98G/118I/208Y/210W/214F/215Y/228R/283I	184V	10.1
		11	41K/46V/54V/64V/82A		181C/190A/219R	6.4
		12	41K/46V/54V/64V/82A		181C/184V/190A/219R	5.5
		13	41K/46V/54V/63H/64V/82A		181C/184V/190A/219R	0.9
		14	41K/46V/54V/64V/82A		69N/101E/108I/181C/184V/190A/219R	2.8
		15	41K/46V/54V/64V/82A		101E/184V	1.8
		16	35D/46V/54V/64V/82A		184V	3.7
		17	35D/46V/54V/64V/69Y/82A		101E/108I/184V	1.8
		18	35D/46V/54V/64V/69Y/82A		74V/101E/108I/181C/184V/190A/219R	1.8
		19	35D/46V/54V/64V/69Y/82A		69N/101E/108I/184V	2.8
		20	35D/46V/54V/64V/69Y/82A		69N/101E/108I/181C/184V/190A/219R	4.6
		21	35D/46V/54V/64V/69Y/82A		181C/184V/190A/219R	4.6
		22	35D/46V/54V/64V/69Y/82A		181C/190A/219R	1.8
		23	46V/54V/64V/69Y/82A		101E/108I/181C/184V/190A/219R	0.9

C	PR90M/46L (5.5)	24	35D/46L/90M	41L/43E/44A/98G/118I/208Y/210W/214F/215Y/228R/283I	181C/190A/219R	0.9
		25	35D/46L/54V/64V/69Y/90M		181C/190A/219R	0.9
		26	36I/41K/46L/90M		184V	0.9
		27	41K/46L/54V/90M		184V	0.9
		28	41K/46L/54V/64V/90M		184V/219R	1.8

D	PR82A/46I (4.6)	29	41K/46I/69Y82A	41L/43E/44A/98G/118I/208Y/210W/214F/215Y/228R/283I	101E/108I/181C/190A/219R	2.8
		30	41K/46I/82A		184V	0.9
		31	35D/46I/54V/82A		184V	0.9

### Non resistance-associated substitutions in PR-RT

Variations also occurred at 4 sites within PR that are not recognized as conferring drug resistance, i.e. P39S, I62V, I64V and I72V. Of these, I64V might be pre-supposed to be of biological significance as a secondary or compensatory mutation, since it was found to cluster together with the 46L, 54V and 82A mutations associated with APV but not 46I and 90M associated with the unboosted IDV and NFV regimens, that were present in 45.8% of the 109 clones analyzed. In the case of RT, prevalent substitutions included V35I, K43E, E122K, S162D, K166R, D177E, E203K, K223Q, L228R, I293V, P294Q, I326V, Q334E, P345Q, A355G, R356K I375V, T376A, T377Q and T400A. A158P, R206S and G359S also appeared at relatively low frequency (i.e. 6%–17%) and in a dispersed pattern, i.e. not as a cluster. By way of comparison, we also analyzed the RNase H region of RT and found that variability was slight, with 3 substitutions present among 90 amino acids at its N terminus in all clones, i.e. L452S, N460D, and N519S (519S is, in fact, the consensus amino acid among subtype B viruses although N519 is present within HxB2). Various frequencies were detected for V466M (24.8%), V467I (20.2%), Y483H (54.1%) and N514S (22%); these substitutions, in fact, are naturally present among various HIV-l subtypes and were probably not selected in the patient. V466M was found to be dispersed yet clustered with Y483H. V4671, Y483H and N514S did not co-exist within any of the clones that were studied. Thus, only 3 to 6 substitutions were identified within RNase H, suggesting that viral evolution had occurred more slowly in this region than in the similarly sized PR region, reflecting differences in drug selection pressure. Although RT inhibitors have been reported to affect RNase H, no mutations in RNase H were identified in our recent report [13].

### Frameshift and nonsense mutations

HIV-1 RT may commonly introduce addition/deletion and/or stop codons into the HIV-1 genome that result, in turn, in an accumulation of defective quasispecies. Our results suggest that defective viral particles may have represented a substantial proportion of viruses within the patient whose samples were analyzed. Of the 109 clones, 6 (5.5%) contained single nucleotide deletions; all of these were located at or near the poly A and C tracts at which RT is believed to be especially error-prone. In-frame nonsense mutations were found at greater frequency, i.e. in 3.7% of cases in PR and in 9.2% of cases in RT. About 18% of the HIV-1 clones analyzed appeared to be defective in PR and/or RT and would be expected to yield non-infectious particles. Defective quasispecies constitute a high proportion of HIV-l particles in infected individuals. Previous reports showed that sequences in uncultured vs cultured PBMCs can be different[14]. 18% of the sequences analyzed in our study were defective in either PR or RT, similar to other reports that examined the *vif *gene[15]. Since the size of the HIV-1 genome is greater than PR-RT, we would expect that the proportion of defective particles produced within an infected individual might be much higher than 18%. Previous results had shown that only one of 10 proviral clones derived from an infected brain sample had the ability to replicate in tissue culture[16]. The significance of defective viruses in HIV pathogenesis should be further investigated.

### Evolutionary relationships among clones

The 109 cloned sequences from PBMCs were genetically related but existed as distinct quasispecies. Only three identical pairs were detected among the 109 clones. Phylogenetic tree analysis of PR sequences suggested that the majority of PBMC quasispecies belonged to 2 distinct groups. Considering the fact that most mutations were synonymous, we further examined protein sequences. In general, the clones could be assigned into 4 groups and 31 subgroups (Table 3). The 82A, 90M, 46I/L, 54V and 64V mutations within PR were important in assignment of the clones to groups, i.e. A-D. Genotyping of plasma viral RNA only detected 82A (Table 1) while proviral DNA clones from PBMCs revealed that about half of them contained either 82A (54.2%) or 90 M (45.8%)(Table 2). These findings indicate the existence of two distinct protease resistance pathways. Table 3 shows that clusters existed among PR46I/90M for group A, PR46L/54V/64V/82A for group B, PR46L/90M for group C, and PR46I/82A for group D, and that 89.9% of the samples belonged to group A (40.4%) or B (49.5%), while groups C and D represented 5.5% and 4.6% of samples, respectively.

Phylogenetic tree analysis of RT suggested that the PBMC quasispecies were closely related, different from HxB2 (not shown). An analysis of drug resistance mutations showed that M184V might exist alone while others mutations usually co-existed with other mutations in the quasispecies (Table 3). The major quasispecies within the PBMCs were subgroups containing PR4IK/46I/90M/RT184V and PR41K/46L/54V/82A/RT184V, and these were represented at 18.3% and 10.1%, respectively (Table 3).

No apparent relationship existed between resistance-associated mutations in the PR vs. RT genes (Table 3), reflecting the fact that they emerged independently during therapy. In contrast, relationships did exist in regard to mutations within either PR or RT. For example, all the PR sequences contained either 82A or 90 M but not both, while 46L/54V/64V clustered mostly with 82A and 46I clustered mostly with 90 M, i.e., groups A and B. Either 41K or 35D, but not both, occurred alongside 90 M or 82A. In the case of RT, 184V or 181C/190A/219R might have emerged independently since both were detected. When combined with the 4 patterns of mutations in PR, i.e. 35D/46I/90M, 41K/46I/90M, 35D/46L/54V/64V/82A and 41K/46L/54V/64V/82A, it appears as though 8 distinct lineages might have existed in this subject from which quasispecies might have been derived. For example, RT181C/184V/190A/219R might have emerged from 181C/190A/219R while RT101E/108I/181C/184V/190A/219R might have emerged from RT181C/184V/190A/219R. In this regard, there was a 3 month interruption in the use of efavirenz (March to June, 1998) that might have contributed to the selective development of the two distinct NNRTI resistance pathways.

The low frequencies of groups C and D suggest that they might have been generated as a result of recombination within the PR region with the cross-over site potentially being between positions 46 and 82. To reduce the possibility of recombination during PCR, we diluted our samples during the second PCR and limited the number of cycles. A control reaction, that employed a mixture of known DNAs, containing wt or mutated PR-RT mutations, revealed that the recombination rate was lower than 3 percent in our experiments (not shown). Several quasispecies re RT might also have been generated through recombination, e.g. RT69N/101E/108I/181C/184V/190A/219R. Variant PR41K/46L/54V/64V/90M could have emerged from PR4IK/46L/54V/64V/82A and 41K/46I/90M crossed over between positions 64 and 82. The RT variant, RT69N/101E/108I/181C/184V/190A/219R could have been generated by the crossing over of RT181C/184V/190A/219R and RT69N/101E/108I/184V between positions 108 and 181.

Infection by HIV is a highly dynamic process, with viral turnover rates being as high as 10^10 ^particles per day[17]. Although PBMCs are probably not the most important site of viral replication in the body[18], they are important in viral spread and represent a source of virus particles that are found in plasma. Hence, PBMCs carry information about the evolutionary process of HIV infection while not necessarily reflecting the majority of viral species at any point in time. Our results are consistent in that we were able to detect certain resistance-associated mutations in plasma, e.g. RT69N, even though this mutation was represented at a level of only 10.2% in cloned sequences and was not detectable in PBMCs by genotyping. The emergence of RT69N was related to the addition of d4T to the treatment regimen. Interestingly, quasispecies carrying PR46I and PR46L were detected at almost equal frequencies in PBMCs (Table 2), and quasispecies containing PR90M and PR46I constituted about half of the viral population in PBMCs but were undetectable in plasma by genotyping. This may be attributed to archival quasispecies identified in PBMCs, as compared to the more recent plasma circulating species.

Dominant quasispecies in plasma represented only a small proportion of those detected in PBMCs, and might reflect the type of drug employed, e.g. RT69N emerged following use of d4T and 3TC (subgroup 16–21 of Table 3). Dominance of PR46L/54V/64V/82A in plasma and disappearance of PR46I/90M from plasma might be related to a switch in treatment from unboosted IDV and NFV to APV, since PR46I and PR90M are primary mutations for IDV and NFV, respectively, but not for APV. The crystal structure of a multiply mutated HIV-1 PR including 46I, 82A, 90M reveals an expanded active site cavity compared to that of wild type[19]. Quasispecies carrying PR46I/90M combined with other mutations, i.e. 10I/16E/37T/48V/63P/77I, are probably not as replication-competent as PR46L/48V/54V/64V/82A under APV pressure.

In our study, wt sequences in regard to PR or RT were not detected in any of 109 clones. However besides 3 identical pairs, 98% (106/109) of these sequences were related but different. All could be divided into about 31 subgroups, with resistance mutations existing in different combinations. The existence of large quasispecies can serve as reservoirs for the emergence of viral species resistant to different drugs, since infected cells might survive for several years [20,21]. Our results on uncultured, infected PBMCs are representative of only a tiny fraction of in vivo infection but indicate an astonishingly high degree of heterogeneity in regard to resistance.

## Authors' contributions

YQ did the experiments, analyzed data and wrote the first version of the manuscript. BGB and MAW supervised the research and revised the manuscript. AD provided all the clinical samples for this work, contributed intellectually to the design of the experiments, and assisted with manuscript revision. All authors read and approved the final manuscript.
